# Obstetrics and Gynecology

**Published:** 1890-03

**Authors:** E. S. McKee

**Affiliations:** Cincinnati


### OBSTETRICS AND GYNECOLOGY.

BY E. S. McKEE, M. D., CINCINNATI.

Asthma Sexuale is the subject of a very interesting and instructive article by Peyer in the Berliner Klinik Heft 9, 1889. Sixteen cases were reported *in extenso*, eleven of which were males and do not concern us in this report. Of the five female patients all suffered more or less from nervous or hysterical troubles which, like the asthma, depended upon the uterine difficulty. In all there was emphysema which caused no great inconvenience. In some of them the sexual act was with such regularity followed by asthma, that the connection between the two was quite clear. The author insists that we should not overlook the importance of the knowledge of the exciting influence of the genital system upon asthma. We should learn if our patients had suffered from nocturnal incontinence of urine. If sexual abuse had been practiced in youth or sexual diseases contracted. The menses, their duration, the molimen, the fluor albus and pain in the lumbar region must be inquired after. If by questioning a leader is gained, then a sexual examination should be asked for. Sexual asthma is naturally not so stubborn as some other kinds. In two young married women, among his cases reported, coitus caused violent attacks of asthmatic sneezing. In another case, the patient suffered from a uterine fibroid with severe asthma, which disappeared after the removal of the tumor. Another was subject to asthmatic attacks which disappeared when she became pregnant. Another suffering from chronic metritis, the cure of the uterine affection relieved the asthma. In all the reported cases, the patients were more or less hysterical and there was a distinct history of neurosis in a family of two of them. The possibility of coincidence should be remembered. It is easy to understand that any aggravation of the uterine or of the ovarian disease and any irritation of the sexual functions might intensify the asthma. The author thinks the treatment of sexual asthma depends upon its origin. The physician should not neglect, through false prudery, to call attention to the bad results of onanism and coitus imperfectus.

Simplification of Cæsarean section is an object to be attained, if not at the expense of security. Fritsch, of Breslau, in the Centrallblatt fuer Gynekologie, XIII 23, 1889, has, as he says, both on therapeutic grounds and to save time, always an object in capital operations, omitted the sero-serous suture of Sanger. He sutures now, as was formerly the custom, by simply passing through the entire uterus wall. The needle is made to enter the peritoneal surface of the uterus about one centimetre from the edge of the wound, and has its exit always about 0. 5-0. 75 centimetres from the border of the wound. The decidua is enclosed in this suture. The sutures must be tied very securely and not more than one centimetre apart. In two cases which he operated on in this manner the results were good. In one the sutures were of silk and sublimate cat-gut. In the other only the latter were used.

Courage and caution in the treatment of abortion is a title which means very much. Just when to be brave and just when to proceed with care is a question which requires not only knowledge and experience but conscience. Of all my recent readings on this subject none have proven so valuable and so well repaid me as the article reviewing the subject during the past year, by Dr. Theophilus Parvin, in the Annual of the Universal Medical

Sciences for 1889, and no one is more the embodiment of cour age and conscience than this author. Écouvillonage, so successfully advocated by Doléris in the treatment of incomplete abortion, has gained much in the eyes of the profession. Strict antiseptic precautions are observed throughout. Frey advocated electricity as a subs itute for the curette in incomplete abortion. For the immediate removal of the retained secundines the faradic current is used, and the galvanic if they have been for sometime retained. Dioviburnia, made by the Dios Chemical Co., in St. Louis, has given excellent results in my hands in those cases where conservative caution was necessary. In one case the patient made desperate attempts to bring on a miscarriage at the seventh and eighth months and seemed about successful, but in both instances the Dioviburnia in dessert spoonful doses, four times a day, tided the patient over her trouble and a fine boy was born at full term.

Papilloma of the female bladder was the subject of a clinical lecture by Dr. C. D. Palmer, at the Cincinnati hospital recently. The patient was a young colored domestic, aged 17 years. She suffered considerable pain, frequent micturition, incontinence of urine, some elevation of the pulse and temperature and amenorrhœa, urethra and bladder very sensitive, and great pain complained of on the introduction of the catheter, urine containing mucus contaminated with blood. The patient was put on the following treatment: Acid boracici dr. i., tr hyosciami oz. ss., infs., buchu oz. iss., M. S., one teaspoonful every three hours. Conjoined manipulation found a tumor on the posterior vesical wall about the size of an almond. The above treatment was continued and the bladder washed out every other day with a saturated solution of boracic acid. The patient improved rapidly and was able to leave the hospital, the tumor having disappeared, about a month after her admission. The doctor had in mind to scrape out the tumor with the spoon if it had not yielded so promptly to treatment. The doctor had another case in which he dilated the urethra with his uterine dilator until the smallest finger of the left hand, then the index finger, could be freely introduced. The tumor was easily found and with the finger nail as a curette it was easily and thoroughly scraped off. Great relief was obtained which, however, did not prove to be permanent. The symptoms gradually returned and in less than one year were as bad as before. About one year after the first operation, cystotomy was performed, an opening being made sufficiently large to freely admit the finger. On the posterior wall of the bladder, about the incision and extending forward to the urethra, were numerous small soft friable projections. These were all broken down and thoroughly scraped with the finger nail and then with the uterine curette. No attempt was made to close the fistulous opening, as it was desirable to give the bladder that complete rest only attained in this way. The bladder was washed out with hot boric acid water solution once a day for a week. Patient got up and about promptly.

Four laparotomies before the class of the Medical College of Ohio, at the Good Samaritan hospital, on four successive clinic days, is, according to the operator, Dr. Reamy, not equalled by any medical college in America. Of these four, one was for the removal of double intra-ligamentous cysts of the broad ligament, one for hæmatocele, one for pyosalpinx and one for hydrosalpinx. The doctor is to be congratulated on his last one hundred cases, with only three deaths. The doctor is a great advocate of the drainage tube, especially if there is a prospect of the secretion of much fluid by the peritonæum. He advises exact coaptation of the peritonæum, checking of all hemorrhage and perfect drying of the cavity of the abdomen. In washing out the abdominal cavity he is careful to elevate the shoulders so as to keep the water off of the diaphragm. He believes in perfect cleanliness. His hands he washes several times on the day of the operation in soap and warm water and carefully cleans his nails while his hands are yet damp. He bathes his hands in bichloride or carbolic acid and lastly in whisky. The instruments are carefully boiled and placed in a solution of carbolic acid and in some instances, just at the commencement of the operation, the knife blade is passed through a gas jet. The abdomen is washed thoroughly and rubbed till it is red. The sponges are treated antiseptically and placed in an alkaline solution, generally bicarbonate of soda. They are used three or four times before thrown away.

